# *EAAC1* gene deletion reduces adult hippocampal neurogenesis after transient cerebral ischemia

**DOI:** 10.1038/s41598-018-25191-4

**Published:** 2018-05-02

**Authors:** Bo Young Choi, Seok Joon Won, Jin Hee Kim, Min Sohn, Hong Ki Song, Tae Nyoung Chung, Tae Yul Kim, Sang Won Suh

**Affiliations:** 1 Department of Physiology, Hallym University, College of Medicine, Chuncheon, 24252 South Korea; 2 Department of Neurology, Hallym University, College of Medicine, Chuncheon, 24252 South Korea; 3 Department of Emergency Medicine, CHA University School of Medicine, Seongnam, 13496 South Korea; 4 Department of Nursing, Inha University, Incheon, 22212 South Korea; 5 Department of Neurology, University of California San Francisco and Veterans Affairs Medical Center, San Francisco, CA 94121 USA

## Abstract

Several studies have demonstrated that excitatory amino acid carrier-1 (*EAAC1*) gene deletion exacerbates hippocampal and cortical neuronal death after ischemia. However, presently there are no studies investigating the role of *EAAC1* in hippocampal neurogenesis. In this study, we tested the hypothesis that reduced cysteine transport into neurons by *EAAC1* knockout negatively affects adult hippocampal neurogenesis under physiological or pathological states. This study used young mice (aged 3–5 months) and aged mice (aged 11–15 months) of either the wild-type (WT) or *EAAC1*^*−/−*^ genotype. Ischemia was induced through the occlusion of bilateral common carotid arteries for 30 minutes. Histological analysis was performed at 7 or 30 days after ischemia. We found that both young and aged mice with loss of the *EAAC1* displayed unaltered cell proliferation and neuronal differentiation, as compared to age-matched WT mice under ischemia-free conditions. However, neurons generated from *EAAC1*^*−/−*^ mice showed poor survival outcomes in both young and aged mice. In addition, deletion of *EAAC1* reduced the overall level of neurogenesis, including cell proliferation, differentiation, and survival after ischemia. The present study demonstrates that *EAAC1* is important for the survival of newly generated neurons in the adult brain under physiological and pathological conditions. Therefore, this study suggests that *EAAC1* plays an essential role in modulating hippocampal neurogenesis.

## Introduction

Adult hippocampal neurogenesis, which originates in neural progenitor cells (NPCs), occurs continuously in the subgranular zone (SGZ) of the dentate gyrus (DG). NPCs from the SGZ migrate a short distance into the granular cell layer (GCL), differentiate, and functionally integrate into the existing neural circuitry, extending axons into CA3 and receiving excitatory synaptic input by sending dendrites into the molecular layer^1,2^. Animal studies have demonstrated that enhancing neurogenesis improves performance in terms of hippocampal-dependent learning and memory tasks^3^. Conversely, reducing hippocampal neurogenesis leads to impaired cognitive function^4^. One key mechanism underlying impaired neurogenesis in aging, stroke, or traumatic brain injury is increased oxidative stress, which serves as a major obstacle for neuronal proliferation, differentiation, and survival. Therefore, the presence of an endogenous cellular defense mechanism against oxidative stress in the above neurogenesis processes has been suggested, and may occur via the enhanced production of endogenous antioxidant molecules. Glutathione, one such molecule, is known to play an important role in maintaining cellular antioxidant function^5^. Although glutathione seems likely to play a role as an antioxidant in neurogenesis, a previous study demonstrated that deletion of the cysteine/glutamate exchanger (xCT), which supplies intracellular cysteine for glutathione synthesis, is not essential for cellular proliferation^6^. Thus, the present study investigated whether intracellular glutathione level is important for stroke-induced neurogenesis in the hippocampus.

Excitatory amino acid carrier type 1 (*EAAC1*, also termed *EAAT3*) was first described as a neuronal glutamate transporter^7^, although it has now been shown to play only a minor role in glutamate removal from the extracellular space, as this task is primarily performed by astrocyte glutamate transporters such as GLT1 and GLAST^8,9^. Several studies have demonstrated that *EAAC1* acts as a cysteine transporter in neurons^10,11^, where it provides a cysteine substrate for glutathione (GSH) synthesis. Aoyama *et al*. showed that loss of the *EAAC1* gene decreased levels of neuronal GSH and increased the degree of oxidative injury during aging, resulting in brain atrophy^12^. Subsequent studies have shown that *EAAC1* gene deletion exacerbates hippocampal and cortical neuronal death after global ischemia^13^ and focal cerebral ischemia^14^. Furthermore, our previous study has demonstrated that N-acetyl cysteine (NAC), a membrane-permeant cysteine prodrug, increases basal GSH levels in *EAAC1*^*−/−*^ mice and reduces ischemic neuronal death^15^. However, the functional role of *EAAC1* in adult hippocampal neurogenesis has not been explored *in vivo* before or after brain injury. Therefore, we investigated our hypothesis that reduced cysteine transport into neurons by *EAAC1* knockout negatively affects adult hippocampal neurogenesis under physiological or pathological conditions. The present study used young or old *EAAC1*^*−/−*^ mice, and cerebral transient ischemia was induced through the occlusion of both common carotid arteries. Histological evaluation for neurogenesis was performed at 7 or 30 days after ischemia was induced.

## Results

### EAAC1 is expressed in both mature and immature neurons

**Figure 1:**
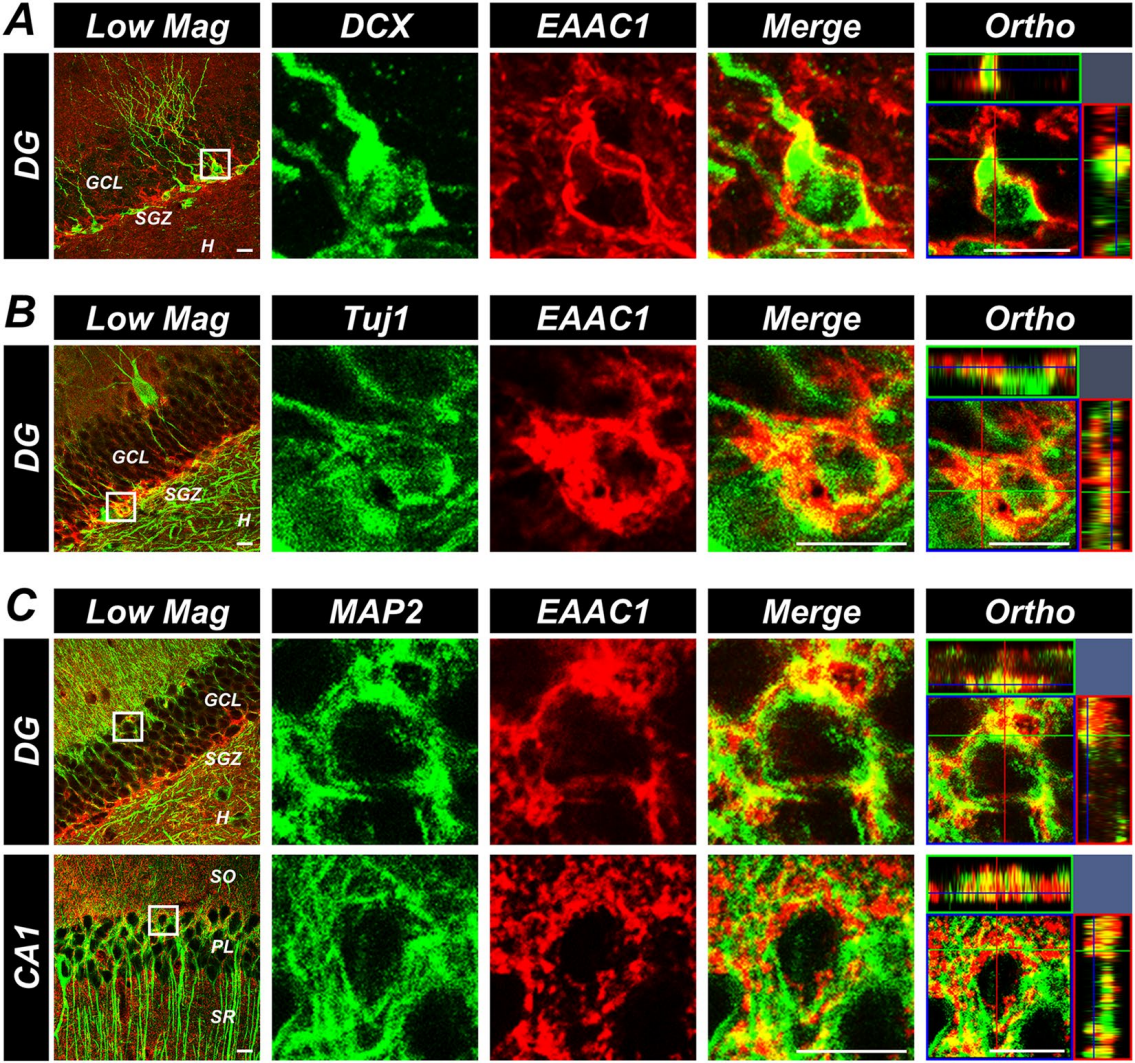
*EAAC1* is expressed in mature and immature neurons. (**A**) Representative immunofluorescence images show DCX (green) and *EAAC1* (red) immunoreactive (DCX+/*EAAC1*+) cells in the DG. (**B**) Representative immunofluorescence images reveal Tuj1 (green) and *EAAC1* (red) immunoreactive (Tuj1+/*EAAC1*+) cells in the DG. (**C**) Representative immunofluorescence images show MAP2 (green) and *EAAC1* (red) immunoreactive (MAP2+/*EAAC1*+) cells in the DG and CA1. Scale bar = 10 μm.

We first investigated whether *EAAC1* is expressed in immature neurons as well as in mature neurons. The presence of *EAAC1* was analyzed using double immunofluorescence staining of *EAAC1* with various neuronal lineage markers, such as DCX, Tuj1, and MAP2. We detected much stronger expression of *EAAC1* in the SGZ of the DG. Double immunostaining of *EAAC1* with MAP2 demonstrated higher expression of *EAAC1* in mature MAP2+ neurons. *EAAC1* is highly expressed in neurons of the central nervous system (CNS)^16^. Consistent with the expression of *EAAC1* in the mature neuron, immature DCX+ and Tuj1+ neurons also displayed expression of *EAAC1* (Fig. 1). These results indicate that *EAAC1* is expressed in both immature and mature neurons.

### Ischemia-induced progenitor cell proliferation is reduced in *EAAC1*^*−/−*^ mice

**Figure 2:**
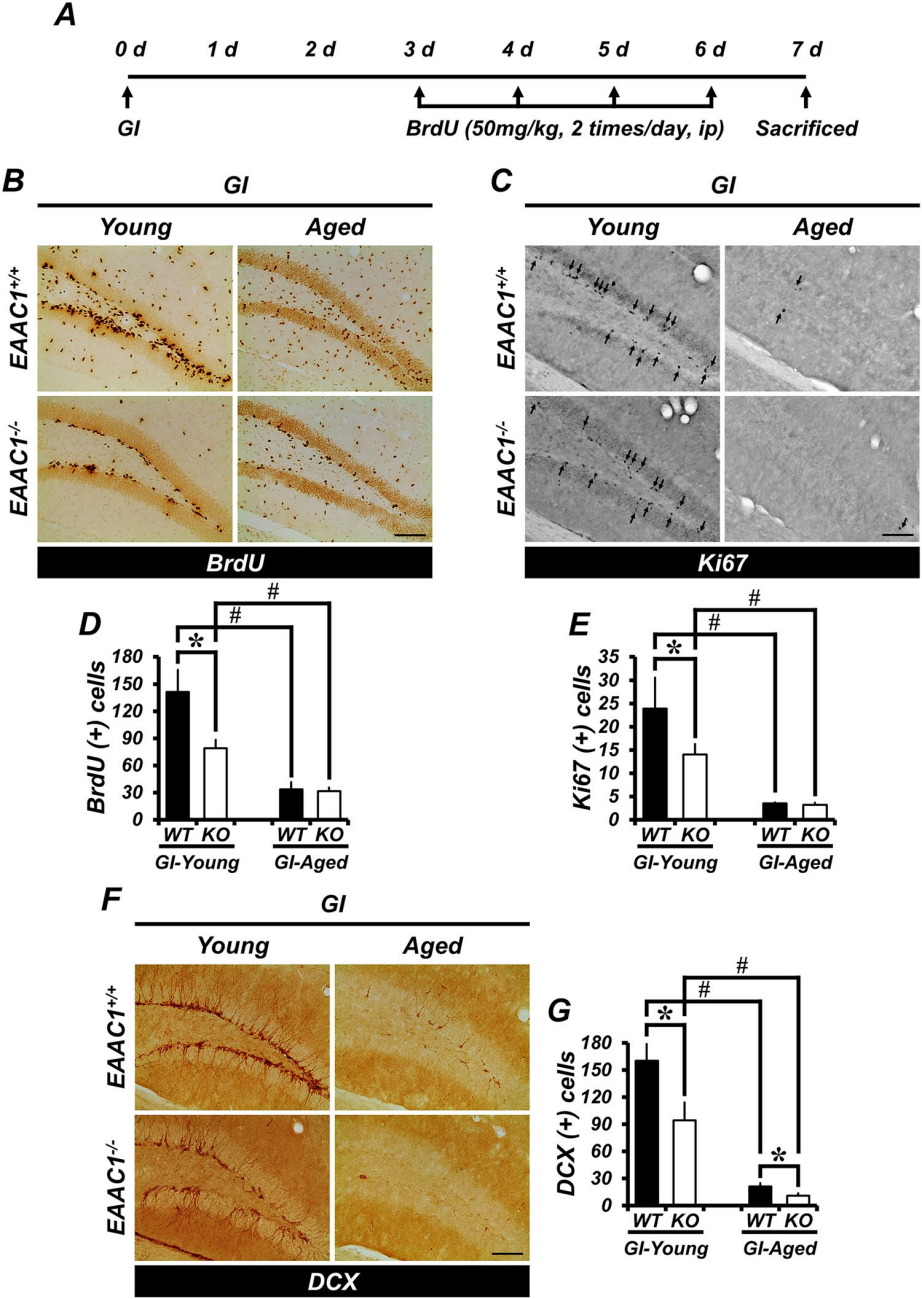
Ischemia-induced progenitor cell proliferation and neuroblast production are reduced in *EAAC1*^*−/−*^ mice. (**A**) The schematic diagram shows the experimental procedure used in this study. BrdU was intraperitoneally injected twice per day for 4 consecutive days from day 3 after ischemia. Mice were killed on day 7. (**B**,**C**,**F**) Representative images reveal BrdU- (**B**), Ki67- (**C**) and DCX-labeled cells (**F**) in the hippocampal dentate gyrus (DG) from young and aged mice (either WT or *EAAC1*^*−/−*^) at 7 days after ischemia. BrdU- and Ki67-labeled cells are located in the subgranular zone (SGZ) of the DG and appear most often in clusters. Detection of BrdU and Ki67 is accomplished with DAB. Ki67-expressing cells are indicated with an arrow. DCX-labeled cells are also located in the inner granular layer. Scale bar = 100 μm. **(D,E G)** Bar graph shows the number of BrdU- (**D**), Ki67- (**E**) and DCX-labeled cells (**G**) in the SGZ from young and aged mice (WT or *EAAC1*^*−/−*^) at 7 days after ischemia. Data are expressed as the mean + SEM; *n* = 7–9 from each group, ^#^*p* < 0.05 versus young mice.

We next sought to determine the effect of deletion of *EAAC1* on the proliferation of progenitor cells in the SGZ of the hippocampal DG after ischemia. Progenitor cell proliferation was analyzed by immunohistochemistry using two different proliferation markers, BrdU and Ki67. Wild-type (WT) and *EAAC1*^*−/−*^ mice were subjected to bilateral common carotid artery occlusion for 30 minutes, and a histological evaluation was performed 7 days after (Fig. 2A). Ischemia led to an increased number of BrdU+ and Ki67+ cells in the SGZ bilaterally in both young (WT, 141.48 ± 23.9 BrdU+ cells, 23.92 ± 6.67 Ki67+ cells; KO, 78.91 ± 9.27 BrdU+ cells, 14.06 ± 2.22 Ki67+ cells) and aged (WT, 33.61 ± 7.76 BrdU+ cells, 3.52 ± 0.21 Ki67+ cells; KO, 31.59 ± 3.84 BrdU+ cells, 3.20 ± 0.42 Ki67+ cells) WT or *EAAC1*^*−/−*^ mice. Interestingly, young mice lacking *EAAC1* showed decreased numbers of BrdU+ and Ki67+ cells after ischemia (Fig. 2B–E).

### *EAAC1*^*−/−*^ mice show reduced neuroblast production after ischemia

We then investigated whether the decrease in ischemia-induced progenitor cell proliferation in the SGZ, caused by gene deletion of *EAAC1*, also gave rise to reduced neuroblast production. The number of DCX+ cells was significantly increased in both young (WT, 160.37 ± 18.48; KO, 94.4 ± 19.58) and aged (WT, 21.0 ± 3.67; KO, 11.5 ± 2.46) WT or *EAAC1*^*−/−*^ mice 7 days after ischemia. In both young and aged *EAAC1*^*−/−*^ mice, the number of DCX+ cells was significantly lower than in young or aged WT mice (Fig. 2F,G).

### Survival of newborn neurons is reduced in *EAAC1*^*−/−*^ mice after ischemia

**Figure 3:**
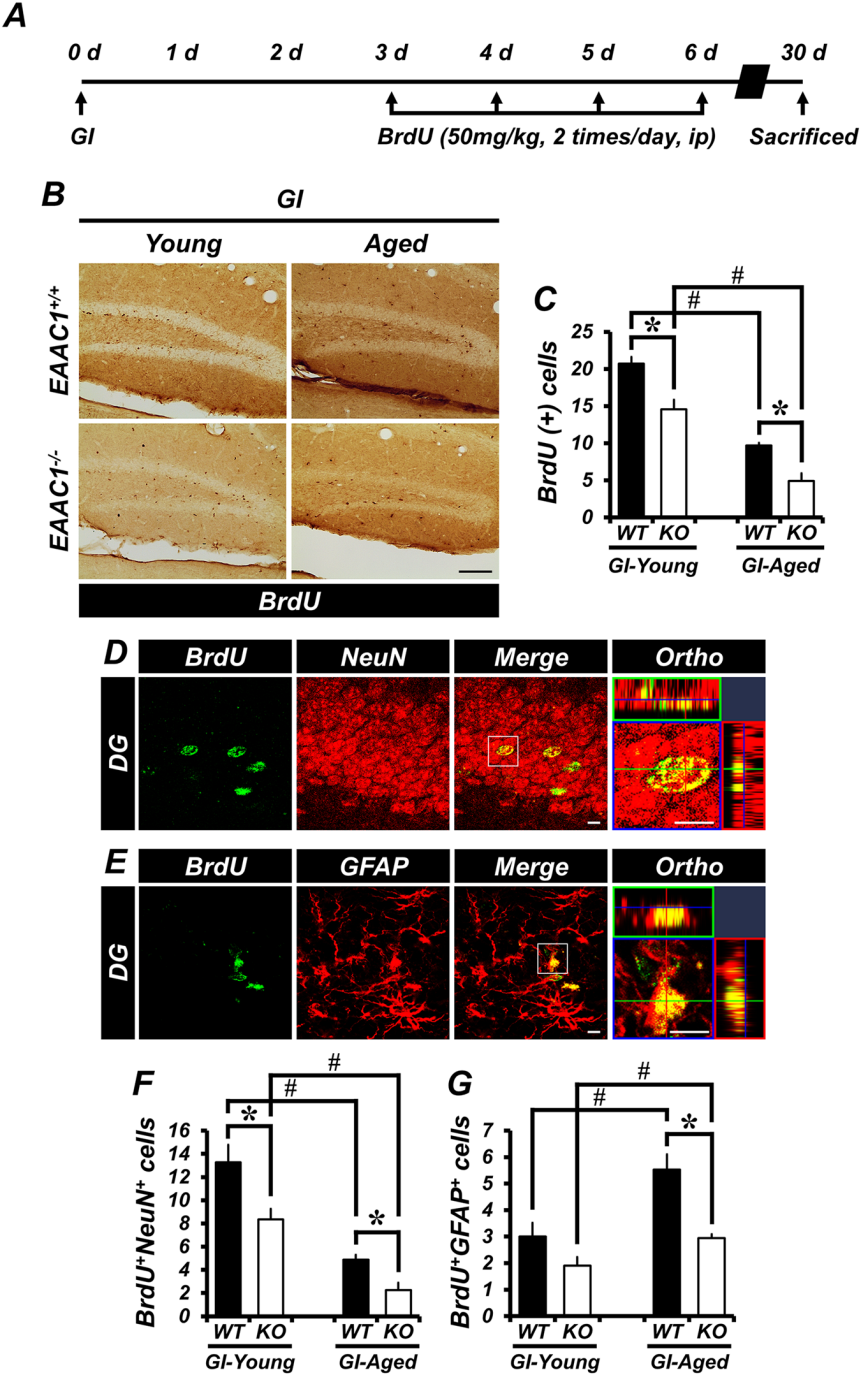
Genetic deletion of *EAAC1* reduces survival of newly generated neurons after ischemia. (**A**) The schematic diagram shows the experimental procedure used in this study. BrdU was intraperitoneally injected twice per day for 4 consecutive days from day 3 after ischemia. Mice were killed on day 30. (**B**) Photomicrographs show the distribution of BrdU-labeled cells in the SGZ/GCL of the hippocampus of young and aged mice (WT or *EAAC1*^*−/−*^) after ischemia. Newborn cells were labeled with BrdU, and their survival was assessed 30 days after ischemia. Scale bar = 100 μm. (**C**) Graph shows the number of BrdU-labeled cells in the SGZ/GCL of young and aged mice (WT or *EAAC1*^*−/−*^) at 30 days after ischemia. Data are expressed by the mean + SEM; *n* = 3–7 from each group, *p < 0.05 versus WT mice; ^#^*p* < 0.05 versus young mice. **(D,E)** Representative immunofluorescence images show the phenotype of cells that have survived in the SGZ/GCL. Scale bar = 20 μm. (**F**,**G**) Bar graph represents the number of BrdU-labeled cells that were colocalized with NeuN **(F)** or GFAP **(G)** in the SGZ/GCL from young and aged mice (WT or *EAAC1*^*−/−*^) at 30 days after ischemia. Data are expressed as the mean + SEM; *n* = 3–7 from each group, *p < 0.05 versus WT mice; ^#^*p* < 0.05 versus young mice.

Although the proliferation of progenitor cells was significantly increased at 7 days after ischemia, the number of surviving newborn cells appeared to return to basal levels 30 days after ischemia. Further, the loss of newly generated cells in *EAAC1*^*−/−*^ mice after ischemia was significantly increased both in the young (WT, 20.72 ± 0.87; KO, 14.56 ± 1.26) and aged (WT, 9.7 ± 0.32; KO, 4.9 ± 1.02) groups, as compared to WT mice (Fig. 3B,C). We assessed the phenotype of cells that survived in the SGZ/GCL at 30 days after ischemia. To this end, double immunofluorescence for BrdU/NeuN or BrdU/GFAP was performed. The total number of NeuN+/BrdU+ cells in *EAAC1*^*−/−*^ mice was significantly reduced in both young (WT, 13.27 ± 1.52; KO, 8.35 ± 0.9) and aged (WT, 4.89 ± 0.41; KO, 2.25 ± 0.64) groups, as compared to WT mice. In addition, the number of GFAP+/BrdU+ cells in *EAAC1*^*−/−*^ mice was similar to that of WT mice in the young group (WT, 3.0 ± 0.51; KO, 1.9 ± 0.33) but was significantly reduced in the aged group (WT, 5.53 ± 0.57; KO, 2.95 ± 0.13). In contrast to the predominantly neuronal differentiation of BrdU+ cells in the SGZ/GCL of the young mice, most of the BrdU+ cells in the aged mice were found to co-localize with GFAP (Fig. 3D–G).

### Deletion of *EAAC1* does not affect the proliferation of basal progenitor cells in SGZ

**Figure 4:**
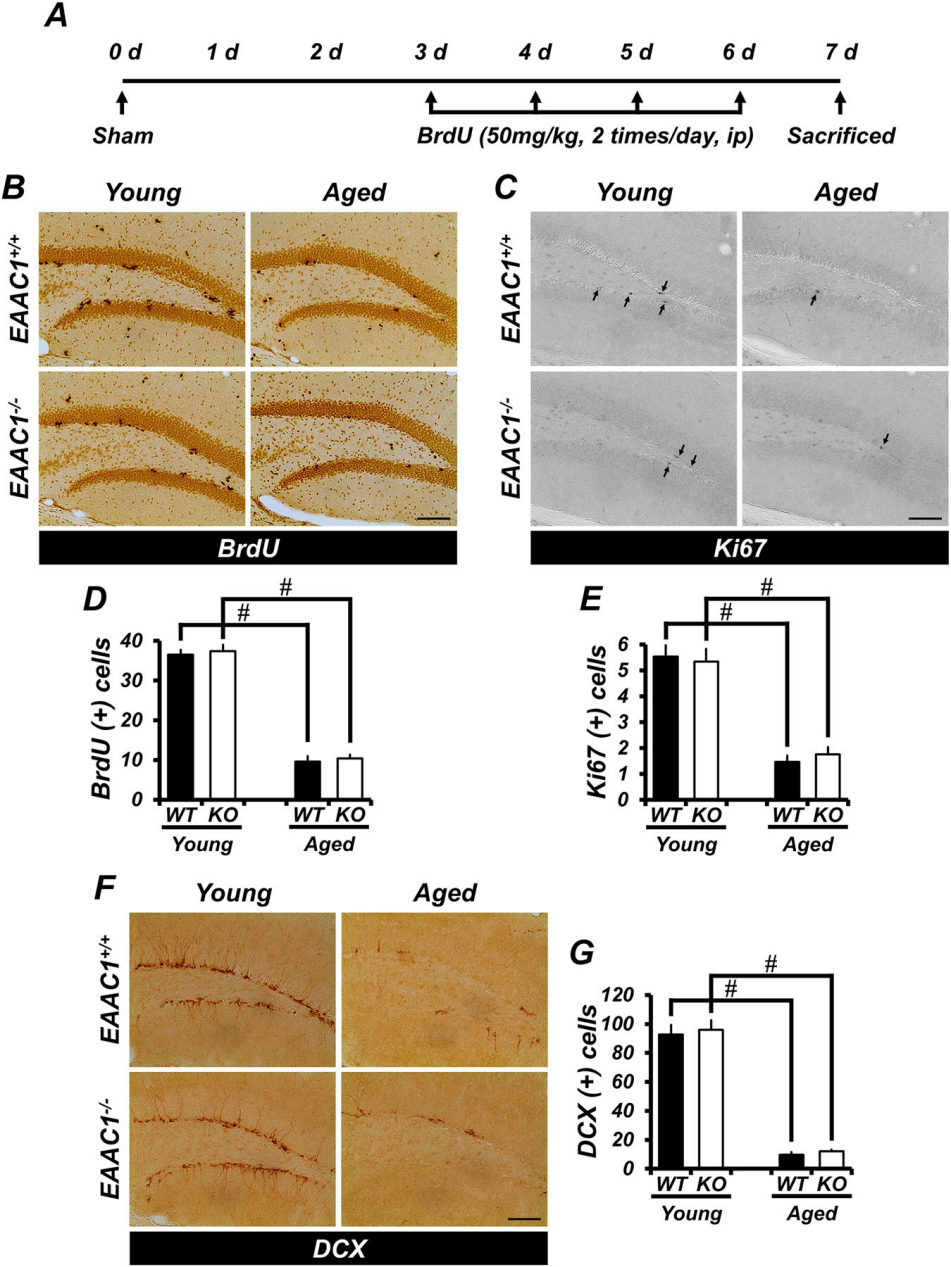
Progenitor cell proliferation and neuroblast production are not different between WT and *EAAC1*^*−/−*^ mice. (**A**) The schematic diagram shows the experimental procedure used in this study. BrdU was intraperitoneally injected twice per day for 4 consecutive days from day 3 after sham surgery. Mice were killed on day 7. (**B**,**C**,**F**) Representative images reveal BrdU- (**B**) Ki67- (**C**) and DCX-labeled cells (**F**) in the hippocampal DG of young and aged mice (WT or *EAAC1*^*−/−*^) at 7 days after sham surgery. The Ki67-expressing cells are indicated by an arrow. Scale bar = 100 μm. **(D,E,G)** The bar graph shows the number of BrdU- (**D**) Ki67- (**E**), and DCX-labeled cells (**G**) in the SGZ from young and aged mice (WT or *EAAC1*^*−/−*^) at 7 days after sham surgery. Data are expressed as the mean + SEM; *n* = 6–10 from each group, ^#^*p* < 0.05 versus young mice.

Next, we investigated whether genetic deletion of *EAAC1* influenced progenitor cell proliferation in the hippocampal subgranular zone (SGZ) of intact brain. The numbers of BrdU+ and Ki67+ cells in the SGZ were remarkably reduced in aged mice, as compared to young mice. However, we did not find any significant differences between wild type (WT) and *EAAC1*^*−/−*^ mice with respect to the number of BrdU+ and Ki67+ cells in either young (WT, 36.5 ± 1.26 BrdU+ cells, 5.7 ± 0.71 Ki67+ cells; KO, 37.37 ± 1.63 BrdU+ cells, 5.39 ± 0.46 Ki67+ cells) or aged (WT, 9.62 ± 1.34 BrdU+ cells, 1.45 ± 0.26, Ki67+ cells; KO, 10.38 ± 0.96 BrdU+ cells, 1.76 ± 0.28 Ki67+ cells) mice (Fig. 4B–E).

### Deletion of *EAAC1* does not affect basal neuroblast production in the SGZ

We also determined whether deletion of *EAAC1* caused an alteration in neuroblast production in the SGZ. For detecting neuroblasts, DCX immunostaining was performed. The number of DCX+ cells in the SGZ was significantly reduced in aged mice compared with young mice. However, no significant differences in the number of DCX+ cells in the SGZ were found between the WT and *EAAC1*^*−/−*^ groups in young (WT, 92.75 ± 6.58; KO, 96.07 ± 6.47) and aged (WT, 9.72 ± 1.63; KO, 12.19 ± 1.03) mice under basal conditions (Fig. 4F,G).

### *EAAC1*^*−/−*^ mice display reduced survival of newly generated neurons

**Figure 5:**
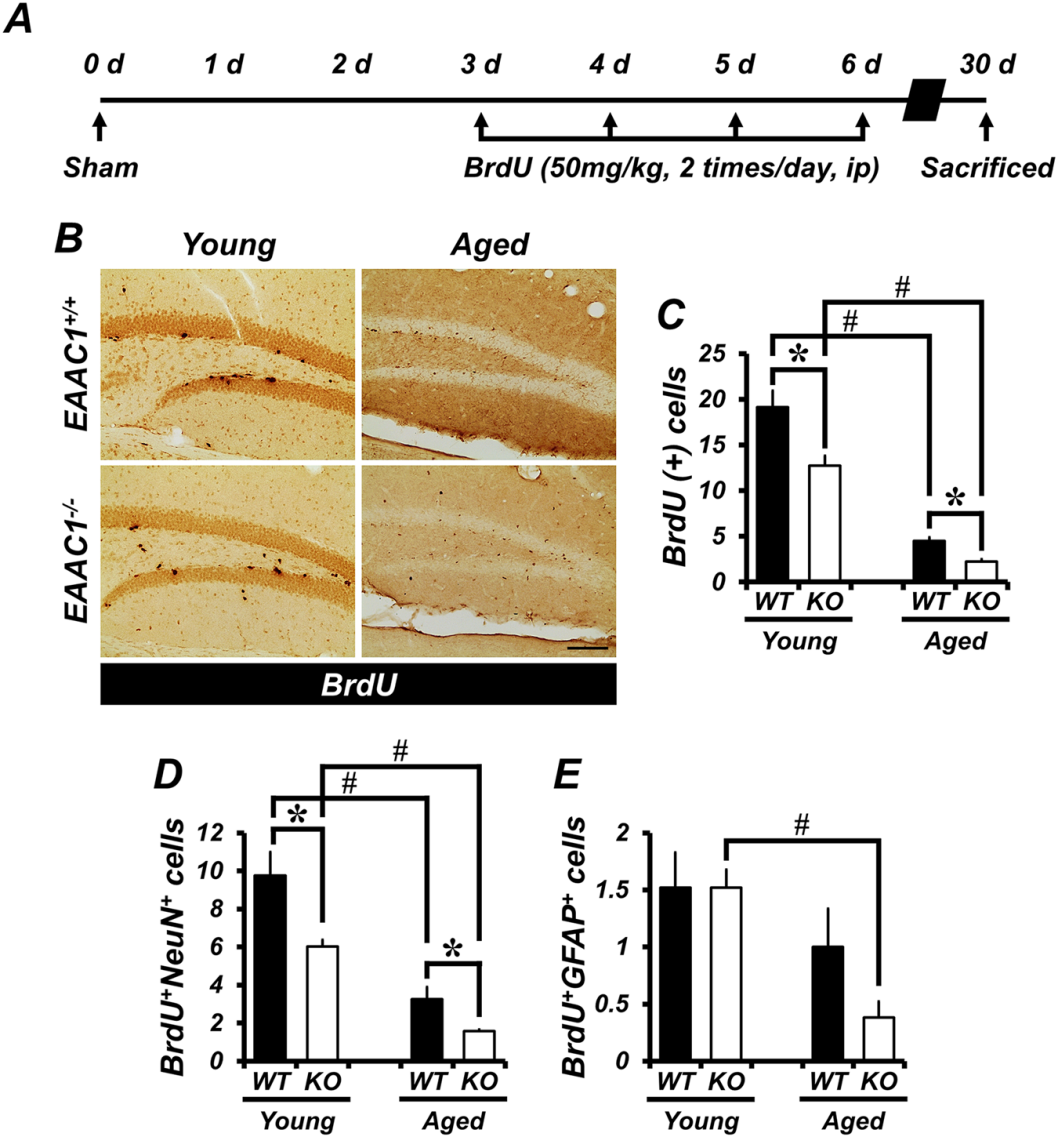
Genetic deletion of *EAAC1* reduces survival of newborn neurons. (**A**) The schematic diagram shows the experimental procedure used in this study. BrdU was intraperitoneally injected twice per day for 4 consecutive days from day 3 after sham surgery. Mice were killed on day 30. (**B**) Photomicrographs show the distribution of BrdU-labeled cells in the SGZ/GCL of the hippocampus of young and aged mice (WT or *EAAC1*^*−/−*^). Newborn cells were labeled with BrdU, and their survival was assessed 30 days after sham surgery. Scale bar = 100 μm. (**C**) Graph shows the number of BrdU-labeled cells in the SGZ/GCL from young or aged mice (either WT or *EAAC1*^*−/−*^) at 30 days after sham surgery. Data are expressed as the mean + SEM; *n* = 6–9 from each group, *p < 0.05 versus WT mice; ^#^*p* < 0.05 versus young mice. (**D**,**E**) The bar graph represents the number of BrdU-labeled cells that were colocalized with NeuN (**D**), or GFAP (**E**) in the SGZ/GCL of young and aged mice (WT or *EAAC1*^*−/−*^) at 30 days after sham surgery. Data are represented as the mean + SEM; *n* = 5–8 from each group, *p < 0.05 versus WT mice; ^#^*p* < 0.05 versus young mice.

Surviving newborn cells, as detected by BrdU immunohistochemistry, were distributed throughout the entire granular cell layer (GCL) at 30 days after sham surgery. Some of those cells had already migrated into the GCL, whereas others were still localized in the SGZ. Quantification of BrdU+ cells in the SGZ and GCL (SGZ/GCL) showed that loss of newly generated cells in *EAAC1*^*−/−*^ mice was significantly increased both in the young (WT, 19.16 ± 1.79; KO, 12.73 ± 1.08) and aged (WT, 4.51 ± 0.38; KO, 2.23 ± 0.28) groups, as compared to WT mice (Fig. 5B,C). We also calculated the ratio of the number of BrdU+ cells in the SGZ/GCL at 30 days to the number of BrdU+ cells in the DG at 7 days after sham surgery and found that the survival ratio of BrdU-labeled cells in *EAAC1*^*−/−*^ mice (young 34.1%, aged 21.4%) was about half that of controls (young 52.5%, aged 46.9; data not shown).

Next, we assessed the phenotype of cells that survived in the SGZ/GCL at 30 days after sham surgery. The total number of NeuN+/BrdU+ cells in *EAAC1*^*−/−*^ mice was significantly reduced in both young (WT, 9.76 ± 1.24; KO, 6.02 ± 0.36) and aged (WT, 3.26 ± 0.64; KO, 1.58 ± 0.08) groups, as compared to WT mice. However, no significant differences were observed between WT and *EAAC1*^*−/−*^ mice in either young (WT, 1.52 ± 0.31; KO, 1.52 ± 0.16) or aged (WT, 1.0 ± 0.34; KO, 0.38 ± 0.14) mice with respect to the number of cells doubly positive for the glial marker GFAP and BrdU (Fig. 5D,E). These results suggest that *EAAC1* is necessary for the survival of newly generated neurons.

### Newly generated neurons suffer from oxidative stress

**Figure 6:**
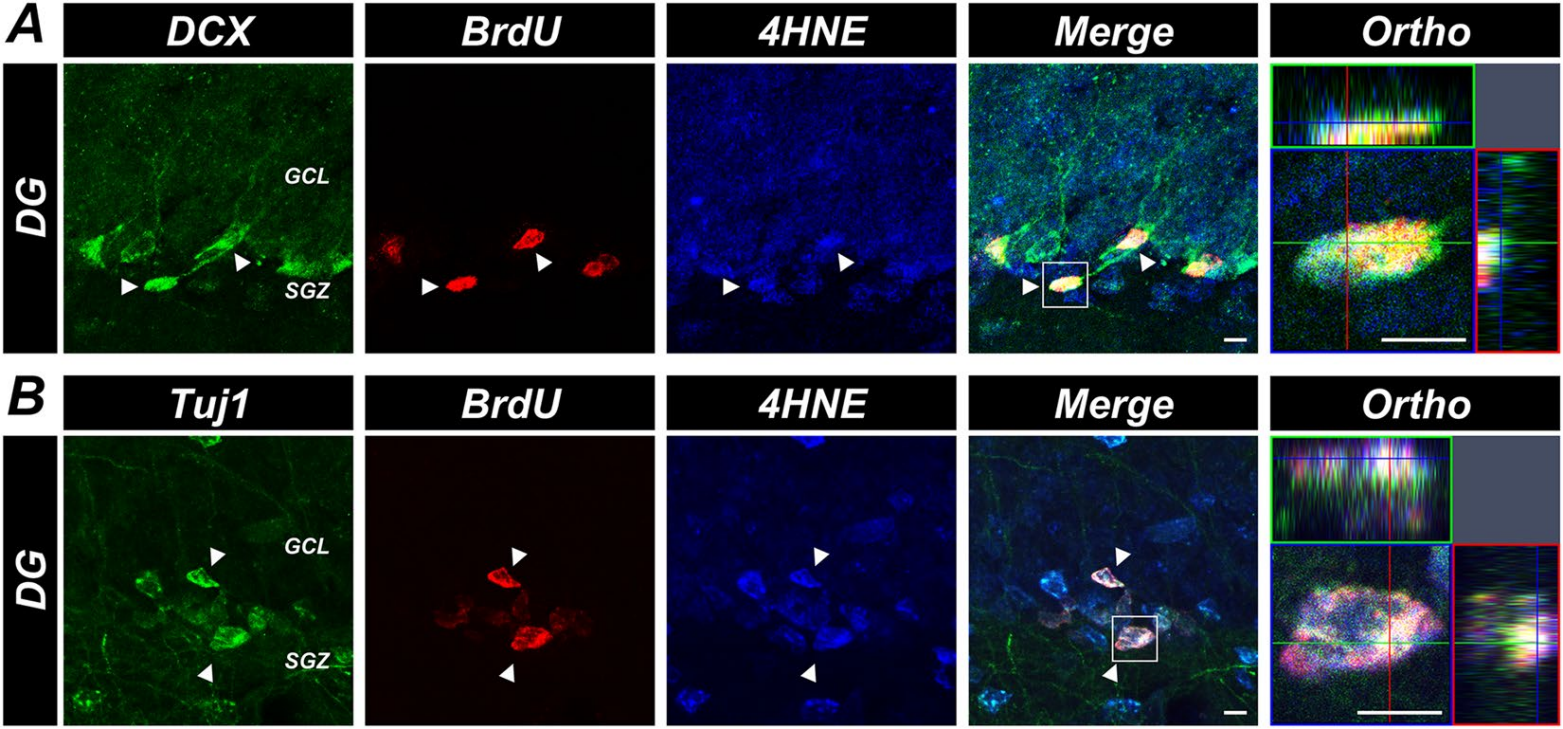
Newly generated neurons are exposed to oxidative stress. (**A**) Representative immunofluorescence images reveal DCX (green), BrdU (red), and 4HNE (blue) immunoreactive (DCX+/BrdU+/4HNE+, arrows) cells in the SGZ/GCL. Scale bar = 10 μm. (**B**) Representative immunofluorescence images show Tuj1 (green), BrdU (red) and 4HNE (blue) immunoreactive (Tuj1+/BrdU+/4HNE+, arrows) cells in the SGZ/GCL. Scale bar = 10 μm.

**Figure 7:**
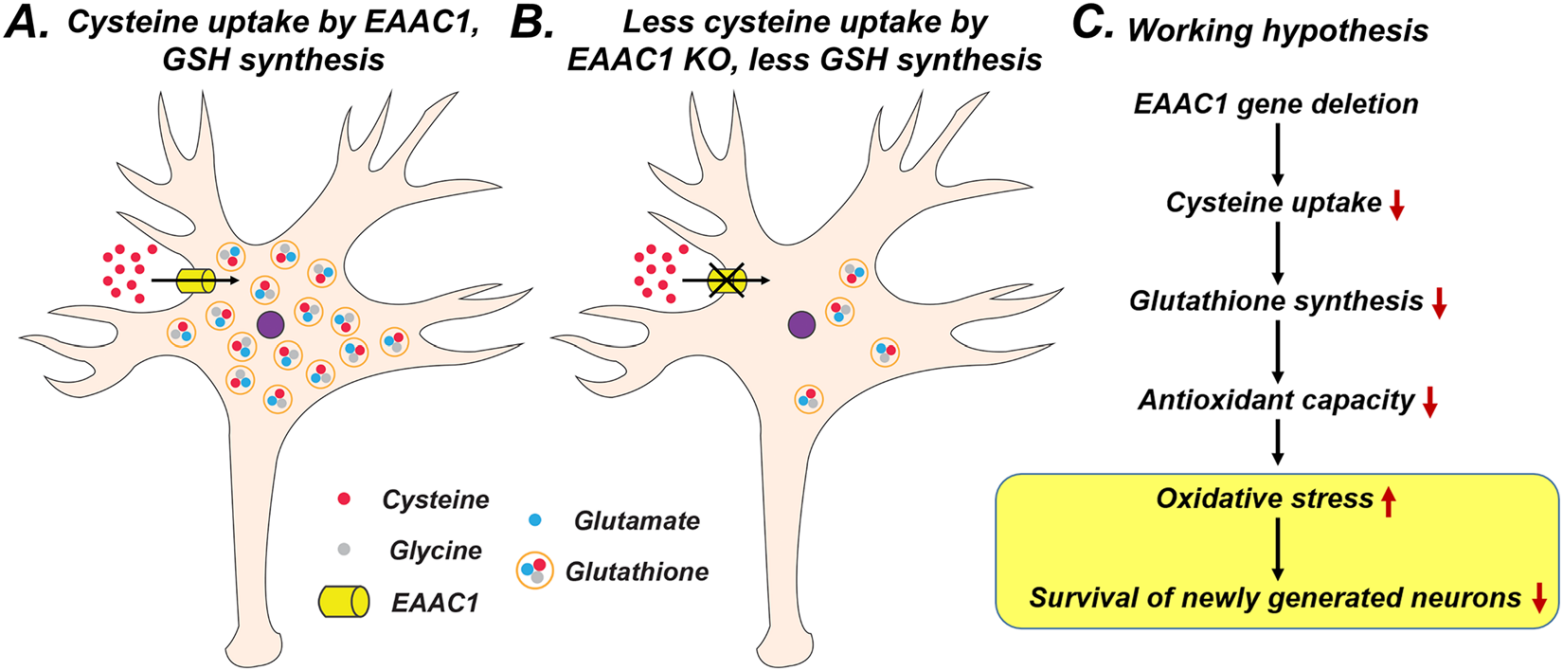
Proposed mechanism by which *EAAC1* knockout reduces the survival of newly generated neurons. This schematic drawing indicates several chain reactions that are thought to occur after impairment of neuronal cysteine transport in *EAAC1*^*−/−*^ mice. (**A**) *EAAC1*-mediated cysteine transport is essential for GSH synthesis in neurons. (**B**) Reduced cysteine uptake by *EAAC1* knockout can result in reduced GSH synthesis. (**C**) Genetic deletion of *EAAC1* attenuates neuronal antioxidant capacity by reduced GSH synthesis. This can create enhanced oxidative stress which subsequently has a negative impact on the survival of newly generated neurons.

We then determined whether newly generated neurons were exposed to oxidative stress. For this, triple immunofluorescence staining for DCX/BrdU/anti-4-hydroxynonenal (4HNE) and Tuj1/BrdU/4HNE was performed. DCX+ /BrdU+/4HNE+ and Tuj1+/BrdU+/4HNE+ cells were seen in the SGZ/GCL. Some of the BrdU+ and DCX+ or Tuj1+ immature neurons were also positive for 4HNE, a major bioactive marker of lipid peroxidation (Figs 6 and 7). These results suggest that newly generated neurons experience periods of exposure to transient oxidative stress.

## Discussion

In the present study, we found that *EAAC1* plays an important role in the survival of newly generated neurons in the hippocampal dentate gyrus. Mice with loss of the *EAAC1* gene displayed normal cell proliferation and neuronal differentiation. However, neurons generated from *EAAC1*^*−/−*^ mice showed poor survival outcomes. The present study also found that deletion of *EAAC1* influenced the entire process of neurogenesis, including cell proliferation, differentiation, and survival after transient cerebral ischemia. These results suggest that cysteine transport through *EAAC1* is crucial for hippocampal neurogenesis under physiological and pathological states.

During adult hippocampal neurogenesis, neural progenitor cells (NPCs) arise from the subgranular zone (SGZ), a region that is located along the border between the granular cell layer (GCL) of the dentate gyrus (DG) and the hilus. To become new neurons, NPCs need to pass through several steps of maturation, such as cell proliferation, migration, differentiation, and survival. However, not all newly produced cells become neurons. A subpopulation develop into glia cells and a substantial number die before complete maturation. Several studies have found that neurons account for the majority of surviving newborn cells, with proportions ranging between 60%^17^ and 93%^18^. However, the present study found that most of the newly generated cells in the aged mice are astrocytes. An explanation for the difference in our findings and previous studies is not immediately clear. One possibility is that NPCs and immature neurons are no longer produced during aging and that active NPCs change into astrocytes after a phase of actively producing NPCs^19,20^. This terminal astrocytic differentiation leads to a decline in NPC populations with increasing age. In addition, Widestrand *et al*. has reported that the reactive gliosis that occurs in the hippocampus of aged mice may be a cause for declining neurogenesis during aging^21^. This evidence describes why the level of neurogenesis decreases with age in mice, while the level of gliogenesis does not^19,22^. The number of mature neurons that finally integrate into the network is strongly dependent on the survival rate of new neurons. Even though many of these newly generated cells die within 4 weeks after birth, a small fraction of them can survive and incorporate into pre-existing neuronal circuits in the hippocampus^23,24^. Therefore, survival of newly generated cells is a critical aspect of maintaining adult hippocampal neurogenesis. The present study found that the number of surviving newly generated cells is reduced in *EAAC1*^*−/−*^ mice as compared to wild type mice, and we suggest that these newly generated cells in *EAAC1*^*−/−*^ mice are more vulnerable to oxidative stress than wild-type mice. We also found that the loss of newly generated cells in *EAAC1*^*−/−*^ mice after ischemia was significantly higher in both young and aged groups as compared to WT mice. We speculate that this may be associated with reduced antioxidative activity due to reduced glutathione levels in these mice.

In the present study, we only found decreased progenitor cell proliferation in young mice lacking *EAAC1* after ischemia. Aging generally causes a several-fold reduction of adult neurogenesis in aged brains as compared to young brains^25^. Kuhn *et al*. suggested several mechanisms that could be responsible for the age-related decrease of neurogenesis: (1) the neural progenitor cells that reside in the SGZ may decrease their proliferative activity; (2) aberrant or inhibited migration could displace the newborn cells; and (3) an increasing number of newborn cells could die before maturation. One of the possible mechanisms for the reduction of neurogenesis appears to result from the inactivation of neural stem cell proliferation. These cells get locked into a quiescent state. Previous studies have also shown that the stem cell pool might get depleted over time, and the massive drop in neurogenesis occurring with aging might be caused by an exhaustion of the stem cell pool^26,27^. Thus, we speculate that the cause of any significant differences between WT and *EAAC1*^*−/−*^ mice in the progenitor cell proliferation in aged mice after ischemia may be related to the depletion of the neural stem cell pool. In addition, although we cannot rule out other mechanisms (such as cell death), a reduction in the proliferative activity of progenitor cells seems to be a major contributor to decreased neurogenesis in aged mice. There are several signaling pathways involved in adult neurogenesis, such as Notch, sonic hedgehog (Shh), bone morphogenetic proteins (BMPs), and Wnts^28,29^. Among them, a reduction in Wnt signaling is related to an age-dependent decline in neurogenesis. Wnts derived from hippocampal astrocytes promote Wnt/β-catenin signaling in neural progenitor cells, leading to neuronal differentiation^30^. Okamoto *et al*. demonstrated that the expression of Wnt-3 and Wnt-3a is progressively decreased in the dentate gyrus during aging, and is followed by a reduction in the expression of NeuroD1^31^. This suggests that the decline in Wnt-3 levels, as well as the number of Wnt-3-secreting astrocytes, is responsible for decreased neurogenesis of the aged brain. Moreover, several studies have suggested that the Wnt signaling pathway is inhibited in the presence of high levels of reactive oxygen species^32,33^. Our previous studies also demonstrated that loss of the *EAAC1* gene causes increased oxidative stress, which leads to brain atrophy during aging^12^ or after ischemia^13^. Thus, we entertain the possibility that the Wnt pathway may be involved in the reduction of neurogenesis in either young or aged *EAAC1*^*−/−*^ mice under physiological or pathological conditions. This speculation should be evaluated through future studies.

Growing evidence suggests that ischemic injury transiently induces a remarkable increase in neurogenesis in both the rodent and primate SGZ. Despite the increase in NPC numbers after ischemic injury, these cells prematurely die without the ability to repair the injured central nervous system. This phenomenon appears to be dependent upon increased levels of oxidative stress. It has been suggested that NPCs may be uniquely predisposed to micro-environmental cues that regulate redox-sensitive pathways to control cellular proliferation after CNS damage^34^, and that oxidative stress is produced as a result of routine adult neurogenesis^35^. Here, we also found that newly generated immature neurons also experience oxidative stress, and this may be implicated in the impaired neurogenesis. In addition, several lines of evidence indicate that antioxidant availability plays a critical role in the survival of neuronal cells in various types of brain injury such as traumatic brain injury^36^, ischemia^37^, seizure^38^, and hypoglycemia^39^. Thus, it seems possible that antioxidant tone could also affect the survival of newly generated cells after ischemia.

Glutathione (GSH) is a tripeptide composed of glutamate, glycine and cysteine. It plays a major role as an antioxidant in the brain^5^. Previous studies have suggested that excitatory amino acid transporters (EAATs) may transport not only excitatory amino acids, such as glutamate and aspartate, but also cysteine^10^. Importantly, *EAAC1* (EAAT3) functions as a concentrative cysteine transporter with an affinity 10- to 20-fold higher than that of GLAST (EAAT1) or GLT-1 (EAAT2)^10,11^. Furthermore, it is well known that EAAC1 expression on the plasma membrane is negatively regulated by glutamate transport associated protein 3–18 (GTRAP3-18)^40^. Aoyama *et al*. demonstrated that *EAAC1* is a critical transporter of cysteine required for GSH synthesis, as evidenced by *EAAC1*^*−/−*^ mice showing both a decrease of neuronal GSH and an increased vulnerability to oxidative stress^12^. Our previous study demonstrated that exogenous treatment with NAC, a membrane-permeant cysteine prodrug, increases neuronal GSH levels in wild-type and *EAAC1*^*−/−*^ mice^15^.

Adult hippocampal neurogenesis might potentially be associated with learning and memory processes. The hippocampus is one of two prominent brain regions where adult neurogenesis takes place, and this region is well defined in the context of learning and memory. In consideration of the decline of cognitive abilities with age, it is noteworthy that hippocampal neurogenesis decreases with age^25,41^. Furthermore, indications for the age-dependent cognitive decline can be reduced by enhanced hippocampal neurogenesis in aged animals^42,43^. Likewise, Gould *et al*. showed that training in specific hippocampal-dependent learning tasks enhanced adult hippocampal neurogenesis^44^. In contrast, several studies found that inhibition of neurogenesis interfered with hippocampal-dependent learning and memory^45–47^. *EAAC1*^*−/−*^ mice also showed age-dependent brain atrophy and cognitive impairment^12^. It is possible that these abnormalities are caused by impaired neuronal GSH metabolism and that loss of the *EAAC1* gene might be related to adult hippocampal neurogenesis (Fig. 7).

Our previous study demonstrated that *EAAC1*^*−/−*^ mice exhibit exacerbated neuronal death and inflammatory responses after transient global ischemia^13^. Thus, observed alterations in the extent of cellular proliferation, production of new neurons, and their survival could be due to a cell-autonomous direct action of *EAAC1* in nascent neurons, or, alternatively, attributable to non-cell autonomous, indirect actions mediated by other cell types. In either case, the fact that survival of mature neurons after ischemia is already compromised in *EAAC1*^*−/−*^ mice indicates that attenuated survival is not selective or unique at all for adult-born neurons in the DG. The lower proliferation rate in *EAAC1*^*−/−*^ mice could also be explained simply by the idea that EAAC1 is involved in survival of stem/progenitor cells in the SGZ under stressed conditions.

Taken together, the present study demonstrates that *EAAC1* is important not only for adult neuron survival but also the survival of newly generated neurons in the adult brain under both physiological and pathological conditions. Thus, this study suggests that *EAAC1* has an essential role in modulating hippocampal neurogenesis and adult neuron survival.

## Methods

### Mouse colony

Animal care protocol and experimental procedures were approved by the Committee on Animal Use for Research and Education at Hallym University (Protocol # Hallym 2014–29), in accordance with NIH guidelines. All experiments were performed in accordance with relevant guidelines and regulations. This manuscript was written in compliance with the guidelines of Animal Research: Reporting *In Vivo* Experiments (ARRIVE)^48^. Male mice from the *EAAC1*^*−/−*^ colony (CD1 strain background) and wild-type (WT) littermates were housed in a regulated environment at 22 ± 2 °C, with 55 ± 5% humidity and a 12:12 hour light:dark cycle with lights on at 8:00 a.m. Mice received a standard diet with Purina (Purina, Gyeonggi, Korea). Food and water were available *ad libitum*. *EAAC1*^−/−^ mice were descendants of the strain established by Peghinni *et al*.^49^, in which exon 1 is disrupted by a neomycin resistance (NEO) cassette. These mice were outbred to wild-type CD1 mice for more than 10 generations prior to these studies. A wild-type (WT) control colony was maintained using the wild-type offspring from the latter outcrosses. WT breeding stock and *EAAC1*^*−/−*^ mice were intercrossed at least once every eight generations to prevent genetic drift, in accordance with the Banbury Conference recommendations^50^. Animals were assigned randomly to transient cerebral ischemia according to an online randomization tool (randomizer.org).

### 5-Bromo-2-deoxyuridine labeling and experimental design

To assess the role of *EAAC1* in adult hippocampal neurogenesis, the thymidine analog BrdU (50 mg/kg; Sigma, St. Louis, MO, USA) was intraperitoneally injected twice per day for 4 consecutive days from the third day following ischemia to label newborn cells. Mice were killed on day 7 or 30 (Fig. 1). For the investigation of progenitor cell proliferation for short-term periods, mice were divided into four groups of young (3–5 months old; weight 25–35 g) and aged (11–15 months old; weight 35–45 g) mice. These groups were comprised of: (1) sham-operated WT mice (young–WT–sham, *n* = 6; aged–WT–sham, *n* = 6), (2) sham-operated *EAAC1*^*−/−*^ mice (young–KO–sham, *n* = 7; aged–KO–sham, *n* = 10), (3) ischemia-induced WT mice (young–WT–GI, *n* = 9; aged–WT–GI, *n* = 7), and (4) ischemia-induced *EAAC1*^*−/−*^ mice (young–KO–GI, *n* = 8; aged–KO–GI, *n* = 9). For the evaluation of cell survival, mice were divided into four groups of young and aged mice, respectively: (1) sham-operated WT mice (young–WT–sham, *n* = 8; aged–WT–sham, *n* = 6), (2) sham-operated *EAAC1*^*−/−*^ mice (young–KO–sham, *n* = 9; aged–KO–sham, *n* = 6), (3) ischemia-induced WT mice (young–WT–GI, *n* = 7; aged–WT–GI, *n* = 3), and (4) ischemia-induced *EAAC1*^*−/−*^ mice (young–KO–GI, *n* = 7; aged–KO–GI, *n* = 4).

### Transient cerebral ischemia

Global ischemia was induced by bilateral common carotid artery occlusion for 30 minutes, as previously described^13,15,51,52^. Mice were deeply anesthetized with isoflurane (1–2% for maintenance; 3% for induction) in a 70:30 mixture of nitrous oxide and oxygen using a isoflurane vaporizer (VetEquip Inc., Livermore, CA). Both carotid arteries were exposed through a midline neck incision. Both common carotid arteries were loosely encircled with a 4/O silk suture before the start of the occlusion. Small aneurysm clips were applied to occlude both common carotid arteries. Wild-type or *EAAC1*^*−/−*^ mice were subjected to bilateral common carotid artery occlusion for 30 minutes under anesthesia with 1% isoflurane. At the end of the 30-minute ischemic period the aneurysm clips were removed, and the common carotids were inspected for normal recovery of blood flow. Following suture of the skin incision, anesthesia was discontinued. When mice showed spontaneous respiration they were returned to a recovery room maintained at 37 °C. Core temperature was kept at 36.5–37.5 °C with a homoeothermic blanket control unit (Harvard apparatus, Holliston, MA). Sham-operated animals received the same neck skin incision under isoflurane anesthesia but common carotid artery occlusion was not performed. Mice exhibiting seizures following ischemia were euthanized and not assigned to data analysis.

### Tissue preparation and immunohistochemistry

Mice were anesthetized by intraperitoneal injection of 1.5 g/kg urethane in sterile 0.9% NaCl at a volume of 0.01 ml/g body weight. A toe pinch was used to evaluate the effectiveness of the anesthesia. Animals were transcardially perfused with 0.9% saline followed by 4% paraformaldehyde (PFA) in phosphate-buffered saline (PBS). The brains were post-fixed with 4% PFA in PBS for 1 hour and then immersed with 30% sucrose for cryo-protection. Thereafter, the entire brain was frozen and coronally sectioned with a cryostat microtome with a 30-μm thickness. To block endogenous peroxidase activity, sections were immersed in 3% hydrogen peroxide for 15 minutes at room temperature. For BrdU immunohistochemistry only, sections were incubated in 2 N HCl at 37 °C for 90 minutes to denature DNA and then rinsed twice for 10 minutes with 0.1 M borate buffer to neutralize the acid. After washing in PBS, the sections were incubated with a rat monoclonal anti-BrdU antibody (diluted 1:150, Abcam, Cambridge, UK) in PBS containing 0.3% Triton X-100 for 1 hour at room temperature. After washing in PBS, the sections were incubated in biotinylated anti-rat IgG (diluted 1:250, Vector, Burlingame, CA) and an avidin–biotin–peroxidase complex (ABC, Vector) for 2 hours each at room temperature. Between incubations, the sections were washed with PBS three times (for 10 min each time). The immune reaction was visualized using 3,3 =-diaminobenzidine (DAB, Sigma-Aldrich Co., St. Louis, MO) in 0.01 M PBS containing 0.03% H_2_O_2_, and the sections were mounted on the gelatin-coated slides. The immunoreactions were observed under an Olympus IX70 inverted microscope (Olympus Co, Shinjuku, Tokyo, Japan). For Ki67 (diluted 1:2k, recognizing nuclear antigen expressed during all proliferative stages of the cell cycle except G0, Novocastra, UK) and doublecortin (DCX, recognizing immature neurons, diluted 1:1k, Millipore, Billerica, MA) immunostaining, incubation with 2 N HCl and 0.1 M borate buffer was omitted.

### Immunofluorescence staining

To identify the phenotype of newly generated cells, double immunofluorescence staining was performed. The immunolabeling procedures were performed as per routine immunostaining protocols. Sections were incubated in mixture of rat anti-BrdU (diluted 1:150, Abcam) with mouse anti-NeuN (diluted 1:500, Millipore) or goat anti-GFAP (diluted 1:200, Abcam) in PBS containing 0.3% Triton X-100 for 2 hours at room temperature. After being washed three times (for 10 minutes each time) with PBS, sections were incubated in a mixture of Alexa Fluor 488 donkey anti-rat IgG (BrdU) with Alexa Fluor 350 donkey anti-mouse IgG (NeuN) or Alex Fluor 594 donkey anti-goat IgG (GFAP) antibodies (diluted 1:250, Invitrogen, Grand Island, NY) for 2 hours at room temperature to visualize primary antibody binding. The sections were washed three times (for 10 minutes each time) with PBS and mounted on gelatin-coated slides. Fluorescence signals were detected using a Zeiss LSM 710 confocal imaging system (Carl Zeiss, Oberkochen, Germany) with a sequential scanning mode for Alexa 488 and 594. Stacks of images (1024 × 1024 pixels) from consecutive slices of 0.9–1.2 μm in thickness were obtained by averaging eight scans per slice and were processed using ZEN 2010 (Carl Zeiss, Oberkochen, Germany).

To determine whether newly generated neurons suffer from oxidative stress, triple immunostaining was performed. We used the following antibodies as primary antibodies: rat monoclonal anti-BrdU (diluted 1:150, Abcam), mouse monoclonal anti-beta 3 tubulin (TUJ1, for recognizing immature neurons, diluted 1:500, Abcam), and rabbit polyclonal anti-4-hydroxynonenal (4HNE, for recognizing oxidative stress, diluted 1:500, Alpha Diagnostic International, San Antonio, TX). For visualization of antibody binding, Alexa Fluor 488-, 594- and 647-conjugated antibodies were applied at a dilution of 1:250 as secondary antibodies.

To test whether *EAAC1* was expressed in immature neurons as well as mature neurons, double immunofluorescence staining was performed. We used the following antibodies as primary antibodies; guinea pig polyclonal anti-DCX (diluted 1:1k, Millipore), mouse monoclonal anti-beta 3 tubulin (TUJ1, diluted 1:500, Abcam), mouse monoclonal anti-microtubule-associated protein 2 (MAP2, for recognizing a neuron-specific cytoskeletal protein, diluted 1:200, Millipore), and rabbit polyclonal anti-excitatory amino acid transporter 3 (*EAAT3*, also known as *EAAC1*, diluted 1:200, Almone labs, Jerusalem, Israel). For the visualization of antibody binding, Alexa Fluor 488- and 594-conjugated antibodies were applied at a dilution of 1:250 as secondary antibodies.

### Quantification

To count the BrdU-, Ki67- and DCX-positive cells, sections were collected at intervals of 180 μm from 1.2 mm to 2.1 mm, posterior to the bregma according to the coordinates of Slotnick and Leonard^53^. Five coronal sections were analyzed from each animals using the microscope with a 10X objective. These sections were then coded and given to a blinded experimenter who counted the number of BrdU-, Ki67- and DCX-positive cells in the SGZ and granular cell layer (GCL) from both hemispheres. Regions of interest were manually drawn over the GCL and SGZ along the superior and inferior blades of the DG (Fig. 1C). For each animal, the numbers of BrdU-, Ki67- and DCX-positive cells in each section were totaled and the results expressed as the mean number of BrdU-, Ki67- and DCX-positive cells per mm^2^ ± SEM. To analyze the phenotype of BrdU-positive cells, as mentioned previously^54^, we determined whether BrdU-positive cells in the SGZ and GCL expressed NeuN or GFAP using confocal microscopy.

### Statistical analysis

Comparisons between experimental groups were conducted using repeated measure analysis of variance (ANOVA) followed by the Student–Newman–Keuls post-hoc test. Data are expressed as the mean ± SEM, and differences are considered significant at *p* < 0.05.

## Electronic supplementary material

Supplemental Figure 1
