# Supplementary material for: EAAC1 gene deletion reduces adult hippocampal neurogenesis after transient cerebral ischemia
Source: Sci Rep. 2018 May 2;8:6903. doi: 10.1038/s41598-018-25191-4 (PMC5932005; doi:10.1038/s41598-018-25191-4)
Supplement: Supplementary file 1 — Supplemental Figure 1 [file 41598_2018_25191_MOESM1_ESM.docx]

***EAAC1* gene deletion reduces adult hippocampal neurogenesis after transient cerebral ischemia**

Bo Young Choi, PhD^1,#^, Seok Joon Won, PhD^5,#^, Jin Hee Kim, PhD^1^, Min Sohn, PhD^4^, Hong Ki Song, MD, PhD^2^, Tae Nyoung Chung, MD, PhD^3^, Tae Yul Kim, MS^1^, Sang Won Suh, MD, PhD^1,^*

^1^Department of Physiology, Hallym University, College of Medicine, Chuncheon, 24252; ^2^Department of Neurology, Hallym University, College of Medicine, Chuncheon, 24252; ^3^Department of Emergency Medicine, CHA University School of Medicine, Seongnam, 13496; ^4^Department of Nursing, Inha University, Incheon, 22212, Korea; ^5^Department of Neurology, University of California San Francisco and Veterans Affairs Medical Center, San Francisco, CA, 94121, USA


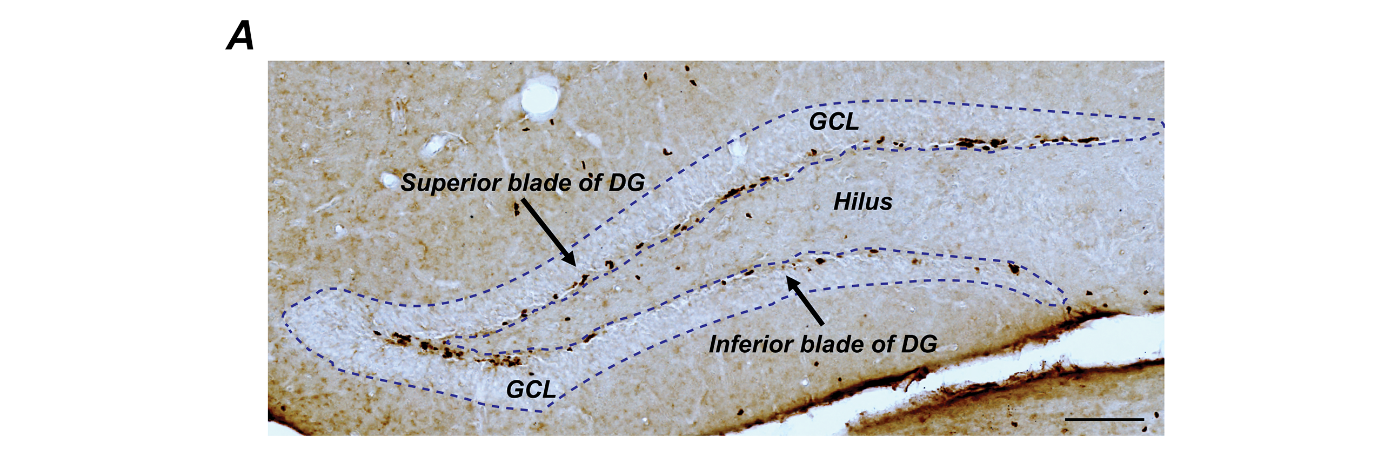


**Supplemental Figure 1. SGZ/GCL of the DG with areas used for quantification.**

**(A)** BrdU-stained coronal section of WT mice 7 days after sham surgery. The area demarcated by the dotted line represents the region of interest used for measurements in the SGZ/GCL.
